# HSV1/2 Genital Infection in Mice Cause Reversible Delayed Gastrointestinal Transit: A Model for Enteric Myopathy

**DOI:** 10.3389/fmed.2018.00176

**Published:** 2018-07-17

**Authors:** Arun Chaudhury, Vijaya Sena Reddy Dendi, Mousumi Chaudhury, Astha Jain, Madhukar Reddy Kasarla, Kiran Panuganti, Gaurav Jain, Abhijit Ramanujam, Bhavin Rena, Sudheer Reddy Koyagura, Sumit Fogla, Sunil Kumar, Nawal Singh Shekhawat, Srinivas Maddur

**Affiliations:** ^1^GIM Foundation, Little Rock, AR, United States; ^2^Christus Trinity Mother Frances Hospital, Tyler, TX, United States; ^3^Wanderful Media/University of Southern California, Los Angeles, CA, United States; ^4^Parkway Surgical and Cardiovascular Hospital, Fort Worth, TX, United States; ^5^Presbyterian Hospital, Denton, TX, United States; ^6^Berkshire Medical Center, Pittsfield, MA, United States; ^7^Woodland Memorial Hospital, Sacramento, CA, United States; ^8^Xenco Laboratories, Houston, TX, United States; ^9^Northwest Medical Center, Bentonville, AR, United States; ^10^Beaumont Hospital, Grosse Pointe, MI, United States; ^11^Neshoba County General Hospital, Philadelphia, MS, United States; ^12^Baptist Hospital, Conway, AR, United States; ^13^All India Institute of Medical Sciences, New Delhi, India; ^14^ESIC Medical College, Sanathnagar, India

**Keywords:** enteric nervous system, nitrergic neurotransmission, virus, guanylyl cyclase, megacolon

## Abstract

In an interesting investigation by Khoury-Hanold et al. (1), genital infection of mice with herpes simplex virus 1 (HSV1) were reported to cause multiple pelvic organ involvement and obstruction. A small subset of mice succumbed after the first week of HSV1 infection. The authors inferred that the mice died due to toxic megacolon. In a severe form of mechanical and/or functional obstruction involving gross dilation of the colon and profound toxemia, the presentation is called “toxic megacolon.” The representative observations by Khoury-Hanold likely do not resemble toxic megacolon. The colon was only slightly dilated and benign appearing. Importantly, HSV1 infection affected the postjunctional mechanisms of smooth muscle relaxation like the sildenafil-response proteins, which may have been responsible for defective nitrergic neurotransmission and the delayed transit. Orally administered polyethylene glycol reversed the gastrointestinal “obstruction,” suggesting a mild functional type of slowed luminal transit, resembling constipation, rather than toxic megacolon, which cannot be reversed by an osmotic laxative without perforating the gut. The authors suggest that the mice did not develop HSV1 encephalitis, the commonly known cause of mortality. The premature death of some of the mice could be related to the bladder outlet obstruction, whose backflow effects may alter renal function, electrolyte abnormalities and death. Muscle strip recordings of mechanical relaxation after electrical field stimulation of gastrointestinal, urinary bladder or cavernosal tissues shall help obtain objective quantitative evidence of whether HSV infection indeed cause pelvic multi-organ dysfunction and impairment of autonomic neurotransmission and postjunctional electromechanical relaxation mechanisms of these organs.

In a recent study, Khoury-Hanold et al. (1) investigates a topic of significant current epidemiological significance viz., genital infection of HSV1, usually an oro-labiotropic virus in mice (1). The authors demonstrate that HSV1 genital infection cause multiorgan autonomic dysfunction, involving the pelvic organs including the urinary bladder and the terminal intestinal tract. The findings are of significant interest, as the study suggests that HSV1 likely affects post-junctional mechanisms of nitrergic downstream signaling that mediate intestinal smooth muscle relaxation.

The authors suggest that transvaginal deliver of HSV1 causes toxic megacolon (1). However, the gastrointestinal phenotype that developed a few days after the infection does not resemble the presentations of “toxic megacolon.” Toxic megacolon is a rare but fatal condition that develops unpredictably and rapidly in certain cases of fulminant colitis. This colitis may result from inflammatory or infectious causes. Rarely, toxic megacolon may develop in hereditary conditions, for example as demonstrated in piebald mice, a model of Hirschsprung disease (2). Megacecum and megacolon often develop slowly in animals including models for Hirschsprung disease (3, 4). Pre-existing inflammation may alter luminal environment and facilitate viral entry, for example like cytomegalovirus in ulcerative colitis (5). Toxic megacolon may develop in colitis developing in concomitant *Clostridium difficile* infection or in subjects with ulcerative colitis (6). This has been examined in animal models of infection and inflammation involving mice, Syrian hamsters and piglets (7). Importantly, systemic toxicity and clinical signs of sepsis are observed, along with massive dilation of colon. In order for reliable pathognomonic diagnosis of toxic megacolon in mice, it is pertinent that two levels of evidence should be presented. First, dilation of small intestinal or colonic dilation must be demonstrated unambiguously, as has been shown in previous studies (Figures 1, 2) (8). Second, evidence of toxicity needs to be presented (Figures 1, 2) (7). Megacolon developing slowly may still show massive dilation of intestines as in Chagas' disease, but does not show local or systemic signs of toxicity (Figure 3) (19). Local signs of toxicity importantly involve ischemia, necrosis or hemorrhage in the wall of the intestine, resulting in unhealthy, angry looking intestinal or colonic loops and obviously, dilation (Figure 1) (7). It may be noted that normal intestinal loops may contain semisolid slurry, not always discrete fecal pellets, or chyme in transit, which may slightly dilate the intestines (Figure 1). This may not be interpreted as megacolon.

**Figure 1:**
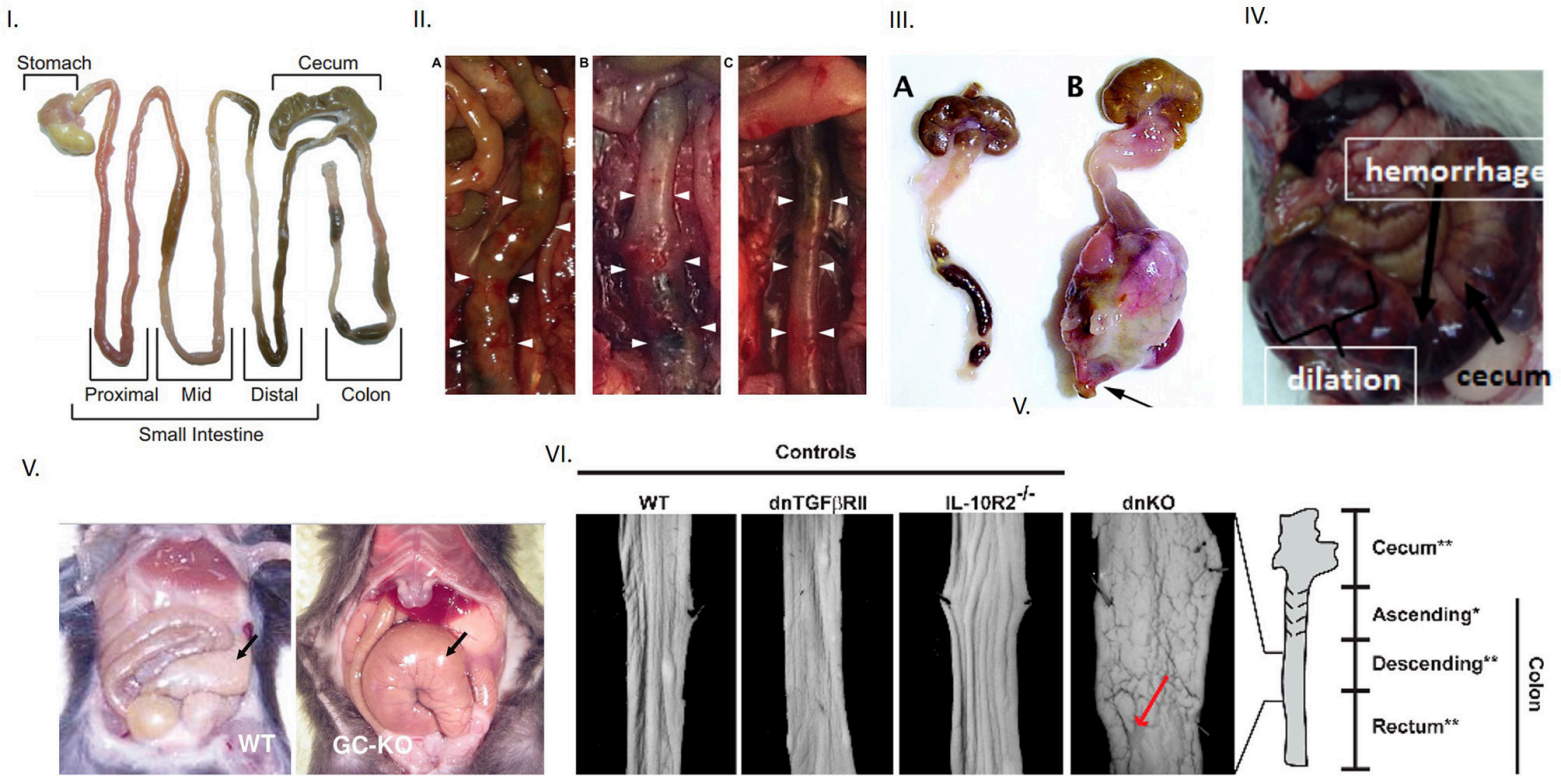
Evidence of toxic megacolon from different mouse models Inference of toxic megacolon requires unambiguous demonstration of septicemia and colonic dilation. **(I)** Appearance of normal mouse intestine Note that intestinal contents may occupy the lumen in both the small and large intestines and slightly inflate the colon, which should not be erroneously inferred as dilation. **(II)** Toxic megacolon in induced chemical colitis in mice by 0.02% BAC The left panel shows dilation at end of 7 days. The middle panel shows frank hemorrhage on intestinal wall after 28 days, in contrast to normal colon in vehicle treated mice seen in the right **(III)** Phenotypic appearance of toxic megacolon The right is a surgical specimen of toxic megacolon that developed in mice after introduction of TGFbeta. Note the massive dilation, but more importantly, the angry-looking intestinal wall, which are matted at places. This results from the ongoing pathophysiology, which may cause inflammation, hemorrhage and necrosis in the wall, and may also cause progressive ischemia, thus causing the intestinal wall to lose its luster. Note that toxic megacolon develops rapidly in a matter of hours or a few days. Note the normal appearing mouse colon on the left panel. It is distended two-folds at places with luminal feces, which should not be interpreted as dilation or retention of feces. **(IV)** Toxic megacolon in pig due to *Clostridium difficile* infection **(V)** An example of slowly developing megacolon Note the dilated, but benign appearing cecum on the right in guanylyl cyclase knockout. The motility disturbances in HSV1 infected mice may have potentially arose from postjunctional defects of guanylyl cyclase signaling. **(VI)** Unambiguous demonstration of colonic dilation Unlike in the single knockouts of models of colonic inflammation, the double knockout mice shows clearly dilated intestines, as evident from the increased perimeter of the laid-open colonic segment. This is a superior way of demonstration of colonic dilation, instead of showing an en face view of the whole intestinal loop (unless there is significant dilation). Reproduced with permission from Koenigsknecht et al. (6), Schmidt et al. (7), Kang et al. (8), Vallance et al. (9), Friebe et al. (10), Khalil et al. (11).

**Figure 2:**
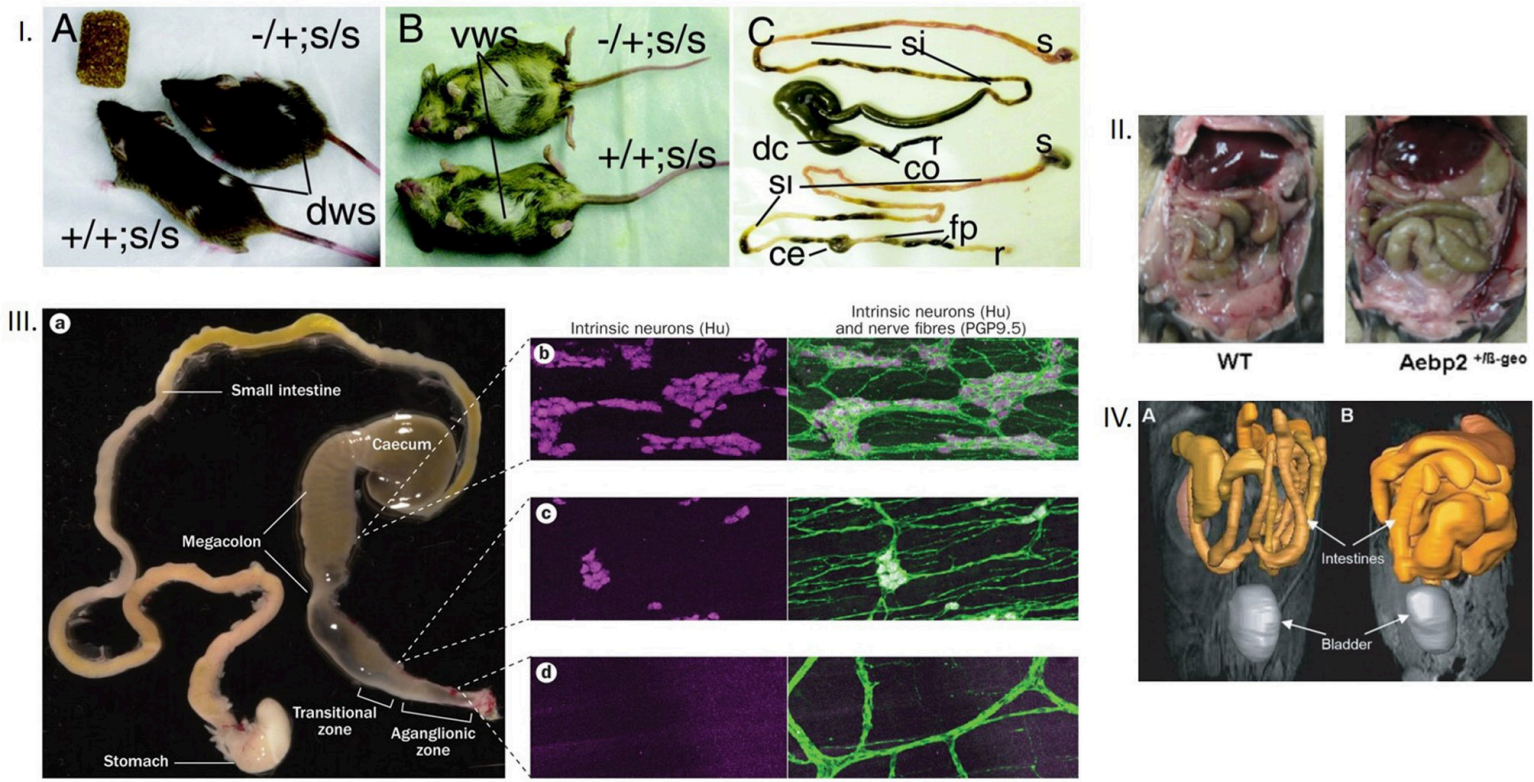
**(I)** Dorsal and ventral appearances of mice with megacolon Note the bulging flank and the abdominal swelling. Also note the comparative enlargement of the cecum and the proximal colon in the mouse GI tract with megacolon. The terminal ileum engorgement could have resulted from the non-functioning cecum, which is way more dilated in megacolon in comparison to the control mouse. The mouse models are Ret-Ednrb mice of megacolon. Ret^−^/Ret^+^; Ednrb^s^/Ednrb^s^ (Upper, model of megacolon) and Ret^+^/Ret^+^;Ednrb^s^/Ednrb^s^ (Lower, control). co, colon, ce, cecum, si, small intestine, s, stomach, fp, fecal pellets, r, rectum. **(II)** Megacolon seen easily after laparotomy Note that in the right panel, another model of megacolon (Aebp2+/b-Geo mice), the loops of dilated colon are easily seen. **(III)** Comparative demonstration of clusters of neurons in myenteric ganglia from the megacolon, transitional and aganglionic zone The megacolon model is of Hirschsprung's disease. Wide areas of the whole mounts are shown for effective comparison. **(IV)** Massive dilation of intestines in an infectious model of megacolon Note the diseased intestines in the right, in comparison to the control in the left. The MRI images in gray scale are color overlaid to demonstrate the intestines. These kind of supporting evidence rigorously conform to inferences of megacolon. Reproduced with permission from Jellicks, (12), Kim et al. (13), McCallion et al. (14), and Obermayr et al. (15) respectively.

**Figure 3:**
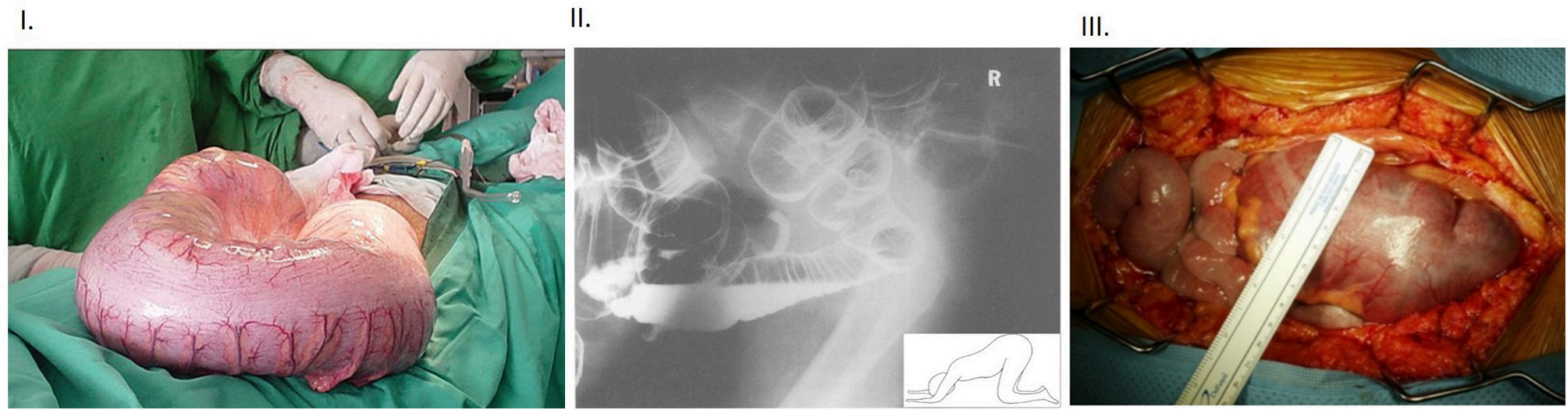
**(I)** Intraoperative view of massive dilation of colon in a subject affected with Chagas' disease Note the benign appearance of the colonic wall despite the massively dilated colon, often seen when the megacolon develops slowly over a period of time. **(II)** Lateral “through-view” imaging of abdomen performed after double contrast barium enema in a patient with toxic megacolon Note that the patient is positioned in knee-elbow position (inset), which is maneuvered to pass out gas. Note the enormously swollen large intestines. The particular position helps the fluid accumulate in the bottom loops of the intestines, whereas the rectum straightens out and slight stimulation, like cough etc., allows the gas to pass out and contribute to decompression of the massively enlarged colon. **(III)** Intra-operative view of toxic megacolon Note the lack-luster appearance of the massively dilated colon and the prominent blood vessels. The colonic swelling may impair the perfusion, and this may have additional effects on extravasation of inflammatory cells. These aspects should be factored in during examination of the inflammatory cells in the muscularis. The “toxicity” criteria in the clinical setting of megacolon is defined as per Jalan et al. (16). Additionally, functional studies like mechanical recordings and evoked potentials may help define the accurate nature of the effects of infection and inflammation on neurotransmission (17). Inflammatory bowel disease and fulminant colitis resulting from infections like *Clostridium difficile* are the common causes of this morbid condition. There are only scant reports of bowel or bladder obstruction from HSV infection (18), though with the changing epidemiology, the correlation needs to be remembered. HSV infection in particular can cause pelvic autonomic neuropathy including constipation and urinary retention as a spectrum of immune reconstitution inflammatory syndrome (IRIS) during management of HIV infection. Reproduced with permission from Munoz-Saravia (19), Panos et al. (20), and Alterman et al. (21).

Khoury-Hanold et al. (1) present incomplete evidence, which does not provide unequivocal demonstration of either megacolon or sepsis. Only a segment of the large intestine with the terminal anus has been shown (1). The upper part of the segment remains filled with colonic slurry. It is possible that the unfilled segment shows the potentially ganglion-depleted segment, which might have arose from the invasion of the myenteric ganglia by HSV1 in that segment. However, the segment containing the colonic slurry is only slightly gorged; this happens normally when chyle from the cecum enters the colon. This type of mild distention is also seen when chyme/chyle transitions through the small intestines during normal intestinal transit. Lack of demonstration of the entire segment of the colon along with the cecum (1) makes the interpretation of megacolon difficult and unconvincing. It is not mentioned whether the colon was dissected open and cleared of feces prior to estimating its weight and normalizing to total body mass. How the study identified the pre-moribund mice is also not clear while estimating the colonic mass. Some of the mice infected with the neuroattenuated strain Δ68H showed decreased survival. In earlier studies of mice models of megacolon, there has been clear demonstration of the aganglionic colon, the transition zone and the proximal segment, which by overwork, massively dilates (Figure 2) (9, 12–14).

In toxic megacolon, the segment of the colon proximal to the obstruction remains massively dilated (mostly with gas, but also with feces), which can be detected easily through radiologic imaging like X-ray abdomen or CT scan (15, 22, 23) (Figure 3). In humans, toxic megacolon involves dilation greater than 6 cm of colon (24). The authors arbitrarily assume a two-fold increase in mass of the colon as their marker for megacolon (1), but it should be remembered that the colon has the intrinsic capacity to dilate to accommodate the contents from the cecum (Figure 1). Demonstration of colonic compliance by demonstrating high volumes at low filling pressures or low colonic tone would be more objective ways to demonstrate true megacolon (25). What is rather important observation is the colonic slurry, rather than formed fecal pellets (1). This may potentially indicate a reduction of colonic mucosal absorptive function, which is possible due to the viral mucositis. Alternatively, this observation could also be normal, as the consistency of the colonic contents show diurnal variation, with scybala (formed pellets) forming only later during the day. Several lines of evidence, if provided like previous studies, would have supported the inferences of “toxicity” and “megacolon”: (i) abdominal distention before laparotomy (ii) angry looking colonic segments (due to massive inflammation) that would have occupied the peritoneal cavity and popped out soon after laparotomy (iii) massively dilated cecum and colon (iv) retained contents in the small intestine (Figures 1, 2). The gastrointestinal transit experiments with FD70 show retention at the level of the small intestine (1). This suggests that the viral spread likely occurred to the small intestine (somewhat supported by the tissue-specific viral titers). If the myenteric ganglia damage was indeed very severe, leading to adynamism, this should have precluded contents from the cecum to enter the colon, as the entire colon would be affected and non-functioning. The only short terminal segment of an unfilled colon (1) suggest a mild functional constipation-like phenotype rather than “toxic megacolon.” Future electrophysiologic studies and smooth muscle mechanical recordings shall help to objectively delineate and quantify the defects in intestinal motility after HSV1 genital infection.

In clinical situations, if the obstruction occurs at the level of ileum in suspected toxic megacolon, it is an ominous situation and emergency (26). For example, if surgical interventions (exploratory laparotomy) are being performed and if dilation or stasis is seen in terminal ileum, it would imply significant obstruction distally, impending colonic perforation and occurrence of subsequent peritonitis and potentially death. The fact that the mice had their obstruction reversed by administration of Miralax (polyethylene glycol) (1), a commonly used osmotic laxative, shows that the delayed transit in the mice after HSV infection was of a functional mild gastrointestinal motility issue of reduced transit, rather than complete obstruction as seen in toxic megacolon or adynamism encountered in intestinal pseudo-obstruction. If toxic megacolon is suspected in the clinical setting, Miralax must not be administered, as it carries the sinister risk of perforating the intestines! Toxic megacolon is a highly critical and dreaded surgical condition and the patients are very sick with high mortality rates (27). Due to irreversible colonic spastic segments, toxic megacolon may rarely be managed medically and often involve extensive surgical resections of the affected intestines (28).

The authors could not precisely delineate the aganglionic or transitional segments (1). It is possible that the ascending kind of HSV infection was stochastic, so different non-contiguous gut segments may be affected. In previous studies of models of megacolon, distinct aganglionic segments have been demonstrated (15). The present study shows representative images of myenteric ganglion in whole mounts that suggests qualitative reduction, but not complete absence (1). Hematoxylin-eosin (H&E) sections showed persistence of myenteric ganglia after HSV infection and development of colonic distention (1). The colonic walls are not thinned out, but rather comparable in thickness between wild type and infected mice. Also, the mucosa do not show any fulminant nature of colitis but intact crypt architecture and only mild mucosal infiltrate, though transmural inflammatory cell infiltrate is visualized in the colon in HSV1 infected mice which had delayed gastrointestinal transit. This type of gastrointestinal mucosal sparing with viral homing to the enteric nervous system has been reported earlier (29).

Profiling the inflammatory immune cells is the strength of this investigative group (1). These tools may be implemented in future studies examining neuroimmune interactions in the enteric nervous system. For example, an important feature in ulcerative colitis (UC) is phenotype reversal, i.e. change of the initial diarrhea-predominant symptoms to constipation (30). In fact, these UC patients are at risk of developing toxic megacolon (22). Thus, endoscopy or endoscopic insufflation is performed with great care in these patients who present with constipation, as the procedure might perforate the colon. There are a few additional points to consider here. The authors hypothesize that neutrophils may be responsible for death of myenteric neurons (1). First, quantitative demonstration of the percent reduction of myenteric neurons has not been provided (1). Neutrophils are seen in the vicinity of the myenteric ganglia (1). However, it may be noted that mobile cells are normally present across the wall of the intestines, though their function is only scantily known (31, 32). It is obvious that some of the HSV1 infected intestinal segments had increased inflammatory cells (1). It is not clear how sick mice were identified during the experiments. For example, how were mice with potentially delayed GI transit identified and randomized to a group that received IgG or the neutrophil blocking antibody αLy6G (1). Interestingly, one of the infected mouse with prolonged luminal transit time survived 7 weeks post-infection, again indicating the mild nature of the pathophysiology involving the enteric nervous system.

The authors suggest that the HSV1 viral particles do not play a direct role in the damage of the myenteric neurons (1), but no direct evidence is provided to this critical assertion. The Circos diagram shows the expression of different viral gene transcripts (1). One of them is the ~140 kB pUS9, whose protein translate functions to interact with kinesin and egress viral particles out of neuronal cells (33). This may be the reason why the viral particles are seen in the extra-junctional place outside the myenteric musculomotor nerve terminal, in addition to within the neuronal cells and processes (1) (Figure 4). Force generating proteins and cytoskeleton are key to regulation of enteric neurotransmission (34–36). So, it is possible that HSV viral particles hijack these cell machineries like other viral particles like rabies and varicella zoster and cause myenteric neuropathy. Whether HSV particles interact with kinesin and dynein for anterograde/retrograde transport through the axons remains to be tested.

**Figure 4:**
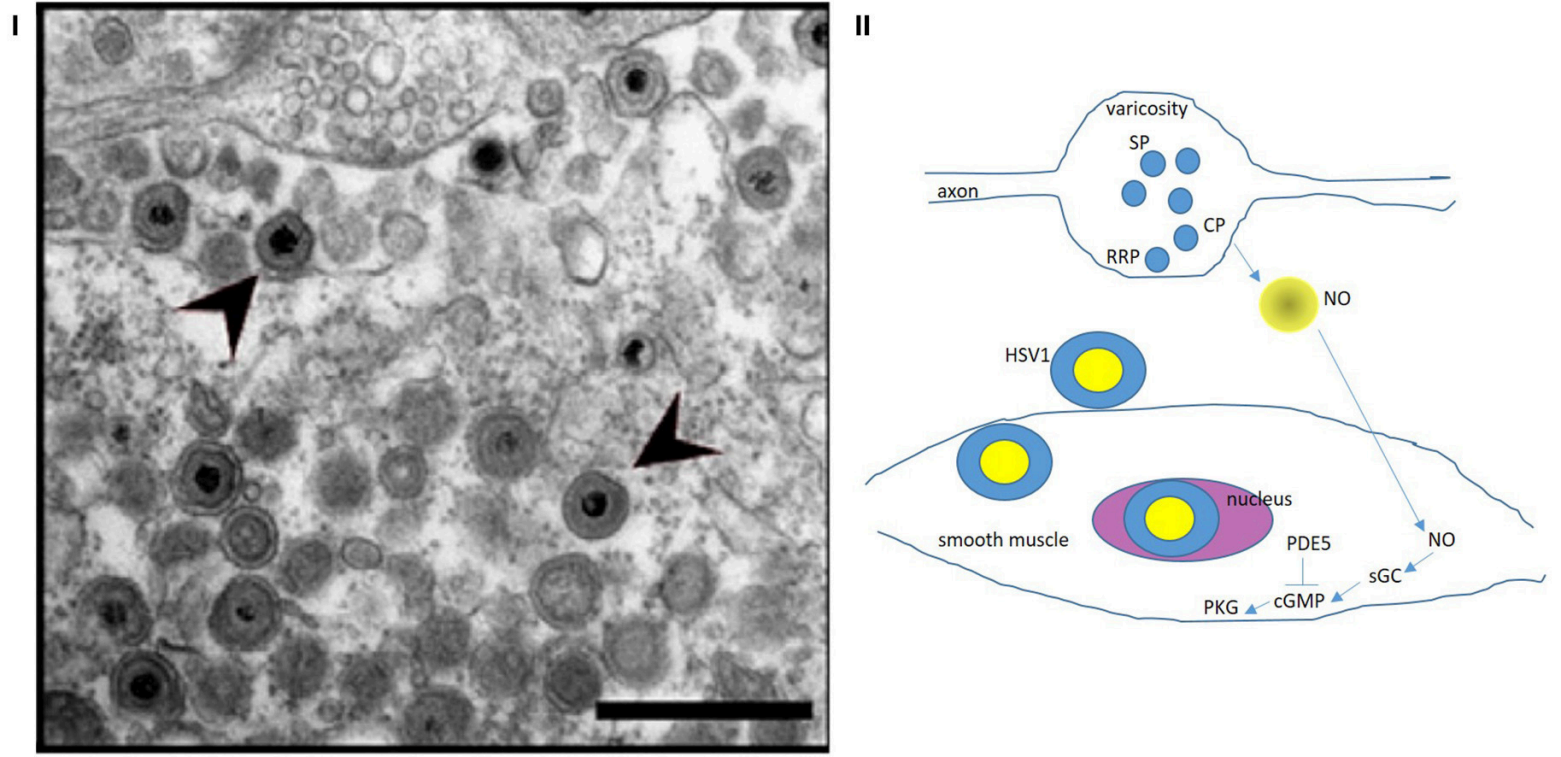
**(I)** Zoomed-out electron micrograph of a mouse with delayed gastrointestinal transit after genital HSV1 infection Note the intact nerve terminal and axon segment in the top (12 o' clock) of the panel. This varicosity shows vesicles docked at the terminal membrane (readily releasable pool, RRP), as well as recycling circulating pool (CP) and storage pool (SP). This likely indicates a normal nerve terminal. This may explain why the HSV1 infection of the enteric nervous system resulted only in a mild phenotype. Also note the HSV1 viral particles in the extracellular space exterior to the terminal. These viral particles probably transgress through the junctional space and enter the post-junctional smooth muscles and may cause defects in the post-junctional proteins like guanylyl cyclase that responds to evoked nitric oxide from the nerve terminals. **(II)** A cartoon depicting the possible pathways that HSV particles may affect and cause smooth muscle myopathy HSV1 affects the sildenafil response proteins, which affect cGMP concentrations. NO released *de novo* from prejunctional terminals mediate smooth muscle relaxation via upregulation of cGMP by soluble guanylyl cyclase. These pathways may be affected by viral integration in the smooth muscle genome, thus impairing smooth muscle relaxation. Colonic, urinary bladder and cavernosal smooth muscles may all be affected, resulting in impaired function of these important pelvic organs. Reproduced with permission from Khoury-Hanold et al. (1). The egress of the viral particles may be facilitated by viral proteins interacting with the neuronal cytoskeleton. NO, nitric oxide; sGC, soluble guanylyl cyclase; cGMP, cyclic guanosine monophosphate; PDE5, cGMP-specific phosphodiesterase inhibitor (sensitive to sildenafil); PKG, protein kinase G.

The authors report the interesting fact that the “sildenafil response proteins” are decreased in the smooth muscles and demonstrate viral particles within the smooth muscles of the muscularis externa (1). These “sildenafil response” proteins prevent degradation of the nitric oxide generated cyclic GMP (cGMP) and enhance activity of its downstream effectors, the key response elements to enteric nitrergic neuromuscular neurotransmission (37–39). It is increasingly identified that inhibitory nitrergic neurotransmission is the final common pathway mediating gastrointestinal motility (35–40). Thus, it is no surprise that the constipation like phenotype due to HSV1 infection may arise due to impairment in the myenteric nitrergic neurotransmission pathways by affecting both prejunctional and post-junctional mechanisms. Vesicularly released ATP mediates the gastrointestinal smooth muscle relaxation along with *de novo* synthesized nitric oxide (38). HSV has been reported to affect ATP mediated neurotransmission by upregulating CD73 (41), an ectonucleotidase that degrades extracellular junctional ATP. The transcellular spread of the viral particles from the enteric neurons to the colonic smooth muscles through the extracellular space may also explain why HSV particles may affect intra-abdominal solid organs like the pancreas and cause pancreatitis (42). The transcellular travel may also facilitate spread across the pelvic space, bypassing the paravertebral or dorsal root ganglia (43).

The authors have made significant endeavor to understand the spread of the HSV1 in the pelvis and beyond (1). Because of intestinal pseudo-obstruction as well as urinary bladder outlet obstruction (1), it seems that there is retrograde transport of the virus through sensory neurons to the region of the sacral plexi, where the viral particles likely cross over and spread to multiple pelvic organs through the sacral parasympathetic outflow neurons. Understanding the mechanisms of these transneuronal pathways is an important step ahead. It remains unclear why the dorsal root ganglion remained relatively unaffected. The present study shows that the axon-pathfinding molecules are downregulated in the gastrointestinal muscularis. A component of axonal pathfinding molecule, netrin, is the main receptor for HSV1 viral entry into vaginal mucosa, and causative for encephalitis (44). The downregulation of netrin in the present study (1) could be an adaptive reaction to the ongoing infection. These discrepancies need to be sorted in future studies. It also remains unclear why only a small subset of mice develops intestinal stasis, and also how many of them developed bladder outlet obstruction. Food and water intake, as well as urine/fecal output of the mice during the advanced stages of illness has not been reported in the present study. It has earlier been reported that HSV can enter the spinal cord through all kinds of peripheral nerves and spread via trans-synaptic spread, cause an ascending disto-proximal demyelinating disease and ascend proximally to the brain, causing encephalitis (45, 46). The dynamics and extent of this spread may be multifold: the strength of the viral inoculum, the spread mechanisms, the local immune defense and the neuroviral interactions preventing lytic cycle or favoring reactivation. In the clinical setting, if there is a presentation with bladder obstruction, obstipation and signs like the vesicles of genital herpes, lumbar puncture is always indicated, as these patients are at high risk of HSV encephalitis (45, 47, 48). There has been no earlier report that these patients had mortality from toxic megacolon related to the HSV infection. Thus, epidemiologically, the gastrointestinal (GI) side effects reported in the current *in vitro* studies (1) may be rare in the actual clinical scenario. Urinary retention due to HSV infection is also rare, though has been reported (47, 49). The bladder outlet obstruction, due to backflow, may have impaired renal infection, caused dyselectrolytemia and death in these mice, rather than sepsis (1). No postmortem appearance of the entire gastrointestinal tract has been shown for the subset of mice who were dead after about 2 weeks of the induced HSV infection. The evidence that the mice succumbed to sepsis due to toxic colonic dilation has not been provided in the present report (1). Features such as free air or fluid in the peritoneum, or hypotension/hypothermia (Figure 1), would have been more supportive of evolving sepsis from severe obstruction and potential perforation of gastrointestinal luminal transit.

The topic of the current investigation is of huge epidemiologic significance, viz. the pathophysiologic consequences of HSV1 genital infection *per se*. The general concepts regarding HSV infection is HSV1 causes oral infection (oral herpes), whereas HSV2 causes genital herpes. The authors rightly bring out the increasing significance of the HSV1 causing genital infections (1, 50). This may be due to changing tropism of the viruses, as well as prevalent sexual practices like “putting mouth on the genital area of the sexual partner,” thus causing exchange of local viruses (40, 51). Importantly, these viral infections can flare up in conditions of suppressed immunity, including pre-existing HIV (human immunodeficiency virus) or other STIs (sexually transmitted infections), pregnancy, use of corticosteroids or immune suppression due to diabetes, treatment of cancer, post-transplantation or auto-immune diseases (52–54). Key areas to explore in future shall include the extent of HSV1/2 infections on disease pathophysiology under controlled conditions of immune compromise (54, 55). Studies have shown that enterocolitis that develops in chronic megacolon like Hirschsprung's disease importantly involve changes in luminal microbiome and mycobiome (56–58). An important area to consider is whether HSV infection may alter luminal microbes and fungal composition, and whether that may be causative of enteric neuronal dysfunction.

All classes of herpesviridae have been reported to infect myenteric neurons, including varicella zoster, cytomegalovirus and herpesvirus, including HSV1 and HSV2 (59–62). In such respect, the present study by Khoury-Hanold et al. (1) do not really usher a novel aspect. There have been some recent reports of viral diagnostic methods which can identify these organisms (63–65). However, the entire field of direct viral detection is in its infancy and these are not routinely employed in the care setting. There are reports of viruses that persist in the myenteric neurons and cause major gastrointestinal dysmotility, for example bornavirus and achalasia (66). These associations have also been reported with HSV (67). However, very little is known regarding the mechanisms of the latency of the viruses within the neurons, their genomic integration or their clearance mechanisms by the immune system and ultimate effect on enteric neurotransmission (68). In immunocompromised states, the “viruses may show their true color.”

The gastrointestinal tract has a massive immune surveillance system due to the challenges of microbes consumed through ingested food. Increasingly, with sexual preferences like sodomy practiced during MSM (men sex with men), the outlet of the gastrointestinal tract is faced with an ever-increasing challenge of tackling microrganisms including viruses (40, 69). Most often, the distal immune system will tackle these infectious agents. But viruses may “walk back” through neurons and thereafter spread far and wide, wreaking havoc. When tell-tales of herpes infection like vesicles are seen, it helps in instituting antiviral prophylaxis and treatment. However, atypical and subclinical presentations can occur, and herpes may manifest without vesicles (46, 70). The present study demonstrates that both HSV 1 and 2 cause neuropathy in pelvic organs (1). Future studies can systematically examine the effect of viruses on nerve densities in the wall of the intestine and any frank morphologic abnormality, or whether the disease is “functional” in manifestation. Both these strains of viruses are neurotropic and can track back to the central nervous system via multiple routes (1, 46), causing life-threatening infections. The autonomic neuropathy can cause multisystem involvement and significant impairment of organ function like delayed gastrointestinal transit and impaired outflow of urine. In our opinion, this significant report of their elaborate investigations by Khoury-Hanold et al. (1) brings forth this hugely important epidemiologic issue into the limelight of systematic scientific investigation.

## Author contributions

AC: conceptualized and drafted manuscript; literature review; VD, MC, AJ, MK, KP, GJ, AR, BR, SRK, SF, SK, NSS, SM: planning of manuscript and clinical and technical collaborative discussions. All authors approved final version of manuscript.

### Conflict of interest statement

The authors declare that the research was conducted in the absence of any commercial or financial relationships that could be construed as a potential conflict of interest.
